# Sociodemographic disparities and contextual factors in obesity: updated evidence from a National Survey of Risk Factors for Chronic Diseases

**DOI:** 10.1017/S1368980021004924

**Published:** 2022-12

**Authors:** Sonia Alejandra Pou, Maria Del Pilar Diaz, Guillermo Angel Velázquez, Laura Rosana Aballay

**Affiliations:** 1 Instituto de Investigaciones en Ciencias de la Salud (INICSA), Universidad Nacional de Córdoba, Consejo Nacional de Investigaciones Científicas y Técnicas (CONICET), Facultad de Ciencias Médicas, Córdoba, Argentina; 2 Estadística y Bioestadística, Escuela de Nutrición, Facultad de Ciencias Médicas, Universidad Nacional de Córdoba, Córdoba 5016, Argentina; 3 Instituto de Geografía, Historia y Ciencias Sociales (IGEHCS), Consejo Nacional de Investigaciones Científicas y Técnicas (CONICET), Universidad Nacional del Centro de la Provincia de Buenos Aires, Tandil, Buenos Aires, Argentina

**Keywords:** Overweight, Multilevel analysis, Nutrition surveys, Built environment

## Abstract

**Objective:**

To assess the association of sociodemographic and environmental factors with the obesity occurrence in Argentina from a sex- and age-comparative perspective and a multilevel approach.

**Design:**

Cross-sectional study based on secondary data from the National Survey of Chronic Diseases Risk Factors (CDRF) 2018, Argentina. Two-level logistic regression models stratified by sex and age were used.

**Setting:**

The nationwide probabilistic sample of the CDRF survey and twenty-four geographical units.

**Participants:**

16 410 adult people, living in Argentine towns of at least 5000 people, nested into 24 geographical units. Sex and age groups were defined as young (aged 18–44 years), middle-aged (45–64 years) and older (65 years and older) men and women.

**Results:**

Single men (all age groups) and divorced/widowed men (aged 45 years or older) had a lower obesity risk compared to married ones. In the middle-aged group, men with higher education showed a lower risk than men with incomplete primary education. In young women, a marked social gradient by educational level was observed. A low-income level coupled with highly urbanised contexts represents an unfavourable scenario for young and middle-aged women. Having a multi-person household was a risk factor for obesity (OR = 1·26, *P* = 0·038) in middle-aged women. Contextual factors linked to the availability of socially constructed recreational resources and green spaces were associated with obesity among young adults.

**Conclusions:**

Socio-environmental determinants of obesity seem to operate differently according to sex and age in Argentina. This entails the need to address the obesity epidemic considering gender inequalities and the socio-environmental context at each stage of life.

Obesity, defined as abnormal or excessive fat accumulation that may impair health^(1)^, is a major public health concern worldwide. The obesity epidemic, although widespread, has been defined as complex in the Latin America and the Caribbean region, where both occurrence and trends vary within the territory and across social groups^(2–4)^. In Argentina, official reports show that the obesity prevalence in the urban adult population has risen from 14·6 % in 2005 to over 25 % in 2018.

Epidemiological research on obesity has traditionally focused on the role of individual-level behavioural factors (diet and physical activity, mainly). Subsequently, greater attention has been paid to social and contextual influences on obesity, since the notions of ‘obesogenic environments^(5,6)^’ and ‘nutrition transition^(7,8)^’ have gradually consolidated.

In particular, the process of nutritional transition implies remarkable shifts in physical activity and diets of the populations with a rapid increase in the prevalence of overweight and obesity. These nutritional changes occur in the context of broader societal drivers (such as sociodemographic changes linked to urbanicity and a rapid economic development), which could mediate differential exposure to the causes of all forms of malnutrition^(9)^. The Social Determinant of Health approach^(10)^ explains that attitudes, beliefs or behaviours represent only the most downstream determinants in the causal pathways influencing health, which are shaped by more upstream determinants; overall, they reflect the economic and social resources and opportunities for improving health^(11)^. Specifically, it has been explained that the upstream determinants that influence obesogenic behaviours may simultaneously manifest as the form of tangible characteristics in the built or natural environments (which determine what is available), as well as less tangible features in our economic, political and sociocultural environments^(12)^. Based on this framework, we assumed that upstream social factors underlie obesity-related behaviours. Additionally, from a multilevel perspective^(13)^, we recognise that certain contextual characteristics of the built and natural environments (defined at a higher level) could operate together with certain individual social features (related to structural mechanisms of social stratification such as income, education, occupation and sex)^(10)^ as socio-environmental factors related to obesity occurrence (defined at a lower level). Especially in developing countries, there is a lack of understanding about the simultaneous individual- and contextual-level factors that drive regional and other subnational disparities (e.g. by sexes and age groups) in obesity burden.

Overall, estimates indicate that the age patterns of obesity differ between men and women^(14)^. Although the ways in which the nutrition transition affects the diets of age groups differently is not well described in the scientific literature, it is recognised that several dimensions of social disadvantage exert differential effects across the life course, impacting on food security and, in turn, on body composition or nutritional status^(15)^. It has been suggested that, for example, the capacity to resist adverse societal influences could play a role in the result of malnutrition – including overweight^(9)^, which could be important among more socially vulnerable population groups (possibly elderly or young women). However, the evidence on the mechanisms explaining age- and sex-related social disparities in obesity is still unclear. In the Latin American and the Caribbean region, the differentials by age groups deserve special attention given their ongoing and accelerated process of demographic ageing^(16)^.

In Argentina, updated and further contextualised evidence is needed to identify socio-environmental determinants of obesity by specific population groups at a national scale. Most of the national population-level studies on adult obesity cover up to the year 2013^(17,18)^; other works on lifestyles and sociodemographic factors related to obesity in this country, though interesting, do not have national representativeness^(18–21)^. Interestingly, some studies have identified diverse socio-environmental patterns and demographic factors accounting for the sex-specific geographical pattern on obesity-related chronic diseases in this country^(22,23)^. Thus, areas with higher mortality risk of CVD in men have been directly associated with the smallest urban scale coupled with a higher level of poverty, in contrast to the effect observed for women^(22)^. Besides, specific geographical patterns with disadvantageous socio-environmental features were identified in Argentina accounting for the differential burden of cancer mortality between sexes in this country^(23)^.

The National Survey of Chronic Diseases Risk Factors (CDRF) has been conducted every 4–5 years since 2005 in Argentina from a probabilistic population-based sample. This survey includes sociodemographic and health information from an urban target population aged 18 years or older living in Argentine towns with at least 5000 people. For the first time, the 2018 edition of this survey included anthropometric measurements (not self-reported data, as previously); the present study analyses the obesity scenario using this representative country dataset. To our knowledge, ours is the first research work that examines up-to-date information on obesity in this country to explain social disparities and contextual factors underlying obesity distribution among different population groups by sex and age. In this population-based study, we assessed the association of individual-level social characteristics and environmental factors, simultaneously, with the obesity occurrence, using a multilevel modelling strategy over the latest available CDRF survey, 2018.

## Methods

### Study design and data sources

This study is based on secondary data collected from the CDRF survey carried out in 2018 by the National Health Ministry of Argentina and the National Institute of Statistics and Census (known by its acronym in Spanish, INDEC). The CDRF is a nationally representative face-to-face survey conducted by trained interviewers, based on a rigorous probabilistic sampling design. The 2018 survey included anthropometric measurements of height and weight taken by trained health personnel. The instruments (portable electronic weighing scale and portable height measuring board) and the techniques used for the height and weight measurements follow the STEPS protocol of the WHO^(24)^ endorsed by the Ministry of Health and Social Development of the Nation, Argentina.

The 2018 CDRF databases consist of a population-based sample of 29 224 persons aged 18 years and older living in towns of at least 5000 people of Argentina. The sampling design of the CDRF was probabilistic and multistage. At the first stage, sampling selection was based on the Master Urban Sample of Dwellings of the Argentine Republic (MUDAR). The MUDAR has a complex sample design and is used by the INDEC as a framework for the selection of private dwellings for all its national surveys. In the CDRF, sampling units were selected from the MUDAR list by means of a stratified probabilistic design (by sociodemographic variables) and a systematic sampling (proportional to the total number of occupied dwellings). For the definitive sample of dwellings in the CDRF, a systematic selection of segments of five contiguous dwellings (within the MUDAR list and cartography) was applied to obtain the final sample of dwellings. At the survey moment, the interviewer selected with equal probability a person aged 18 years or older, assisted by a random algorithm. The final size of the sample was 29 224 individuals for the application of the first part of the questionnaire (self-reported data), covering all jurisdictions in the country. For the second step that includes anthropometric measurements, a probabilistic subsample was made with 75 % of previously selected dwellings (*n* 16 577 individuals). More methodological details of the CDRF survey have been published in official reports of the National Health Ministry^(25)^.

In our study, a subset of 16 410 persons with anthropometric measurements was extracted, after excluding 167 observations with incomplete data. For multilevel analyses, the individual-level dataset (*n* 16 410) was nested into the area-level information about environmental characteristics (*n* 24 geographical units, corresponding to administrative divisions). Two area-level variables were selected: the index of socially constructed recreational resources (SCRR) and the index of green spaces. The SCRR index is a score (calculated at provincial scale) that considers the availability of certain environmental conditions and cultural attractions such as urban aesthetics, urban heritage sites, cultural centres, shopping malls, sports centres, among others. The green space index (score) represents the availability of green spaces measured through land coverage by open green spaces or natural areas. Both indexes were part of the latest available Quality of Life Index database^(26)^, which incorporates data for Argentina at the county level from several sources (official reports, field studies and satellite imagery) into a Geographic Information System. This dataset and its methodological aspects were published elsewhere^(23,26)^.

### Statistical analyses: multilevel modelling

Due to the hierarchical structure (spatial clustering) of the data (16 410 subjects nested into 24 geographical units), two-level logistic regression models were used to estimate the association between selected individual- and area-level covariables and obesity occurrence (dichotomous outcome, yes/no). Three age groups were defined as young (aged 18–44 years), middle-aged (aged 45–64 years) and older (65 years and older) considering the criteria of the INDEC and the MeSH descriptor of middle-aged provided by the US National Library of Medicine^(27)^. Thus, the sex and age groups defined were set up as strata in multilevel model adjustments. Obesity was defined as having a BMI ≥ 30 (yes/no) following the WHO criterion^(1)^. BMI was calculated by using measured anthropometric data.

The analyses were performed in sequential steps, from a variance component model (multilevel ‘empty’ model, without covariates) to an adjusted two-level model that included all the individual- and area-level variables. Model selection was based on the Akaike information criterion as well as the interpretability and comparability of results among the different population groups. Several *individual-level variables* that inform about sociodemographic characteristics of participants were considered in the models: marital status (married; divorced/widowed; single), household type (one-person household; a couple without children; multi-person household including a couple with children or other persons at home), education (highest level of education attained: incomplete primary education or lower; primary education; high school; higher education), income level (higher, intermediate or lower if self-reported incomes are at the highest, second-to-four or first quintile of the income distribution, respectively) and geographic location of residence (city of residence classified by population size as: small cities of 5000–149 999 people, or big/middle-sized cities of 150 000 or more people). Since previous findings have indicated a joint effect of poverty and urbanisation on health statistics in Argentina^(16)^, additive interaction terms between income and geographic location were also considered. Finally, lifestyle-related variables such as physical activity (intense/intermediate/low), tobacco consumption (non-smoker/former smoker/smoker), and fruit and vegetable consumption (above or below the five portions/day recommendation by the Food Guide for the Argentine Population)^(28)^ were included as adjustment variables. These data were gathered by a structured questionnaire administered by trained personnel. This instrument included questions about tobacco consumption (current consumption frequency and previous consumption of at least 100 cigarettes, among others) and the usual frequency of fruit or vegetable intake (times/week and daily portions by self-report), and a section with the International Physical Activity Questionnaire (IPAQ) for physical activity assessment^(29)^.

The *area-level variables* included in the model were the aforementioned contextual indexes (continuous variables at the provincial scale) of SCRR and of green spaces; both were calculated as population size-weighted average of the indexes reported at the county level^(26)^. The highest values for these indexes reflect the best situation of the availability of SCRR or green spaces.

The linear predictor of the general mixed logistic model was

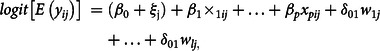




where y_*ij*_ is the response variable (obesity yes/no), x_*1ij*_ to x_*pij*_ and *w*_*1j*_ to *w*_*lj*_ are explanatory variables with fixed effects (linear coefficients), corresponding to the individual- or area-level covariates, respectively. The term *ξ_j_* is a random intercept term representing the clustering variance structure and, therefore, accounting for the geographical variability in the estimation process. All models were adjusted by lifestyle-related variables. Thus, female-only and male-only models were fixed and the estimated association measures (OR) plotted. Then, six models were constructed for each sex and age group combination (young, middle-aged, and older men and women). All analyses were performed using Stata v14.

## Results

This study examined the obesity occurrence among 16 410 adult people (58 % women and 42 % men) living in Argentina, using data from the CDRF survey. In 2018, over half (51·0 %) of the participants were young adults, and about 30·1 % and 18·9 % were middle-aged and older adults, respectively.

Table 1 presents the individual-level characteristics of the study participants, for the total sample and stratified according to age group and sex. As shown, about 46·8 % of the participants reported a low level of physical activity, reaching values of 54·7 % and 61·8 % in older men and women, respectively. The percentage of people with low fruit and vegetable intake was around 90 % in all age groups and both sexes. The percentage of smokers (21·8 % in whole sample) was higher in men than women, especially in those under 65 years of age. In older adults, the percentage of former smokers was more than double in males (45·4 %) compared with the female group (19·4 %). Most participants were married (49·5 %) and lived in a multi-person household (61·2 %). However, the distribution of subjects by marital status or household type shows differences between sexes. These differences were stronger in the elderly and were particularly related to the categories married or divorced (marital status variable), and one-person household (household-type variable). About 36 % of the total sample had completed primary education (with higher prevalence in men than women, especially in the 18–44 years age group), while 17·3 % reported having higher education. In all age groups, the percentage of people with higher education was higher in women than in men. Over half (59·9 %) of the participants had an intermediate income level. Particularly at higher-income levels, men always show higher values (%) compared to women, being the difference more noticeable in younger groups. Regarding geographical location, approximately 55–60 % of participants from all age group and both sexes lived in big/middle-sized cities (Table 1).

**Table 1:** Individual-level characteristics by age group and sex among 16 410 adults. National Survey of Chronic Diseases Risk Factors, Argentina 2018

	Men								Women								Both sexes	
	Young men (aged 18–44 years) *n* 3657		Middle-aged men (aged 45–64 years) *n* 2103		Older men (65 years or more) *n* 1200		Total adult men *n* 6960		Young women (aged 18–44 years) *n* 4714		Middle-aged women (aged 45–64 years) *n* 2839		Older women (65 years or more) *n* 1897		Total adult women *n* 9450		Total adult population *n* 16 410	
	Subjects	% by columns*	Subjects	% by columns*	Subjects	% by columns*	Subjects	% by columns*	Subjects	% by columns*	Subjects	% by columns*	Subjects	% by columns*	Subjects	% by columns*	Subjects	% by columns*
Individual-level characteristics																		
Anthropometric characteristics																		
Weight status by BMI†																		
Underweight	70	1·9	4	0·2	7	0·6	81	1·2	135	2·9	23	0·8	12	0·63	170	1·8	251	1·5
Normal weight	1303	35·6	362	17·2	242	20·2	1907	27·4	2000	42·4	683	24·1	427	22·5	3110	32·9	5017	30·6
Pre-obesity	1363	37·3	878	41·7	508	42·3	2749	39·5	1355	28·7	915	32·2	663	34·9	2933	31·0	5682	34·6
Obesity	921	25·2	859	40·8	443	36·9	2223	31·9	1224	26·0	1218	42·9	795	41·91	3237	34·2	5460	33·3
Lifestyles-related characteristics																		
Physical activity																		
Intense	1042	28·5	344	16·4	119	9·9	1505	21·6	776	16·5	357	12·6	130	6·8	1263	13·4	2768	16·9
Intermediate	1230	33·6	740	35·2	423	35·2	2393	34·4	1842	39·1	1028	36·2	577	30·4	3447	36·5	5840	35·6
Low	1349	36·9	999	47·5	656	54·7	3004	43·2	2064	43·8	1434	50·5	1172	61·8	4670	49·4	7674	46·8
No response	36	1·0	20	0·9	2	0·2	58	0·8	32	0·7	20	0·7	18	0·9	70	0·7	128	0·8
Fruit and vegetable consumption																		
< 5 portions/day	3377	92·3	1939	92·2	1078	89·8	6394	91·9	4331	91·9	2556	90·0	1699	89·7	8586	90·9	14 980	91·3
5 or more portions/day	163	4·5	108	5·1	86	7·2	357	5·1	228	4·8	224	7·9	166	8·7	618	6·5	975	5·9
No response	117	3·2	56	2·7	36	3·0	209	3·0	155	3·3	59	2·1	32	1·7	246	2·6	455	2·8
Tobacco consumption																		
Non-smoker	1961	53·6	931	44·3	508	42·3	3400	48·8	3066	65·0	1804	63·5	1361	71·7	6231	65·9	9631	58·7
Former smoker	595	16·3	597	28·4	545	45·4	1737	25·0	597	12·7	490	17·3	369	19·4	1456	15·4	3193	19·5
Smoker	1101	30·1	575	27·3	147	12·2	1823	26·2	1051	22·3	545	19·2	167	8·8	1763	18·7	3586	21·8
Social characteristics																		
Marital status																		
Married	1724	47·1	1325	63·0	691	57·6	3740	53·7	2385	50·6	1453	51·2	545	28·7	4383	46·4	8123	49·5
Divorced or widowed	185	5·1	454	21·6	404	33·7	1043	15·0	422	8·9	877	30·9	1147	60·5	2446	25·9	3489	21·3
Single	1748	47·8	324	15·4	105	8·7	2177	31·3	1907	40·4	509	17·9	205	10·8	2621	27·7	4798	29·2
Household type																		
One-person household	678	18·5	525	25·0	442	36·8	1645	23·6	518	11·0	636	22·4	961	50·7	2115	22·4	3760	22·9
Couple without children	393	10·7	437	20·8	461	38·4	1291	18·5	449	9·5	488	17·2	384	20·2	1321	14·0	2612	15·9
Multi-person household	2586	70·7	1141	54·3	297	24·7	4024	57·8	3747	79·5	1715	60·4	552	29·1	6014	63·6	10 038	61·2
Education (highest level of education attained)																		
Incomplete primary education or lower	154	4·2	242	11·5	310	25·8	706	10·1	173	3·7	299	10·5	469	24·7	941	10·0	1647	10·0
Primary education	1287	35·2	905	43·0	546	45·5	2738	39·3	1318	28·0	1053	37·1	797	42·0	3168	33·5	5906	36·0
High school	1703	46·6	606	28·8	237	19·7	2546	36·6	2276	48·3	807	28·4	383	20·2	3466	36·7	6012	36·6
Higher education	513	14·0	350	16·6	107	8·9	970	13·9	947	20·1	680	23·9	248	13·1	1875	19·8	2845	17·3
Income level (self-reported family incomes)‡																		
Lower level	843	23·0	385	18·3	118	9·8	1346	19·3	1290	27·4	566	19·9	198	10·4	2054	21·7	3400	20·7
Intermediate level	2082	56·9	1197	56·9	836	69·7	4115	59·1	2678	56·8	1667	58·7	1376	72·5	5721	60·5	9836	59·9
Higher level	732	20·0	521	24·8	246	20·5	1499	21·5	746	15·8	606	21·3	323	17·0	1675	17·7	3174	19·34
Geographical location of residence§																		
Big cities or middle-sized cities	2076	56·8	1253	59·6	687	57·2	4016	57·7	2715	57·6	1643	57·9	1060	55·9	5418	57·3	9434	57·5
Small cities	1581	43·2	850	40·4	513	42·7	2944	42·3	1999	42·4	1196	42·1	837	44·1	4032	42·7	6976	42·5

*The percentage distribution by categories of each variable was calculated on the total subjects (n as the 100 %) within each population group studied.

† Weight status by BMI: underweight (BMI de18·5); normal weight (BMI 18·5–24·9); pre-obesity (BMI 25·0–29·9); obesity (BMI ≥ 30).

‡ Income level: lower level (lowest quintile); intermediate level (middle quintiles); higher level (highest quintile).

§ Geographical location of residence: small cities (5000 up to 149 999 people); big cities or middle-sized cities (150 000 or more people).

A similar distribution across sex and age groups was found for area-level variables. In the whole sample, 33·9 % and 55·1 % lived in areas with high indexes (above the mean values) of the SCRR and green spaces, respectively. The mean (sd) score was 6·30 (0·76) for SCRR index and 1·65 (0·63) for green spaces, with maximum/minimum values of 4·96/9·00 and 0·67/2·94, respectively.

**Fig. 1.**
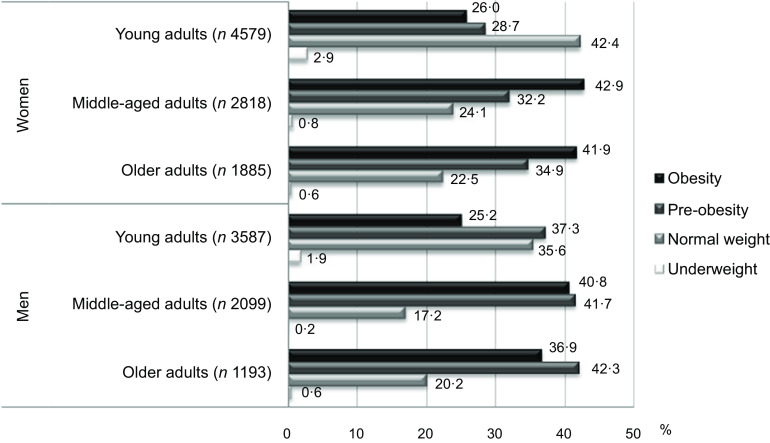
Weight status distribution by age group based on a sample of 9450 women and 6960 men. National Survey of Chronic Diseases Risk Factors, Argentina 2018. Classification of weight status by measured BMI: underweight (BMI<18.5); normal weight (BMI 18.5–24.9); pre-obesity (BMI 25.0–29.9); and obesity (BMI≥30)

**Fig. 2.**
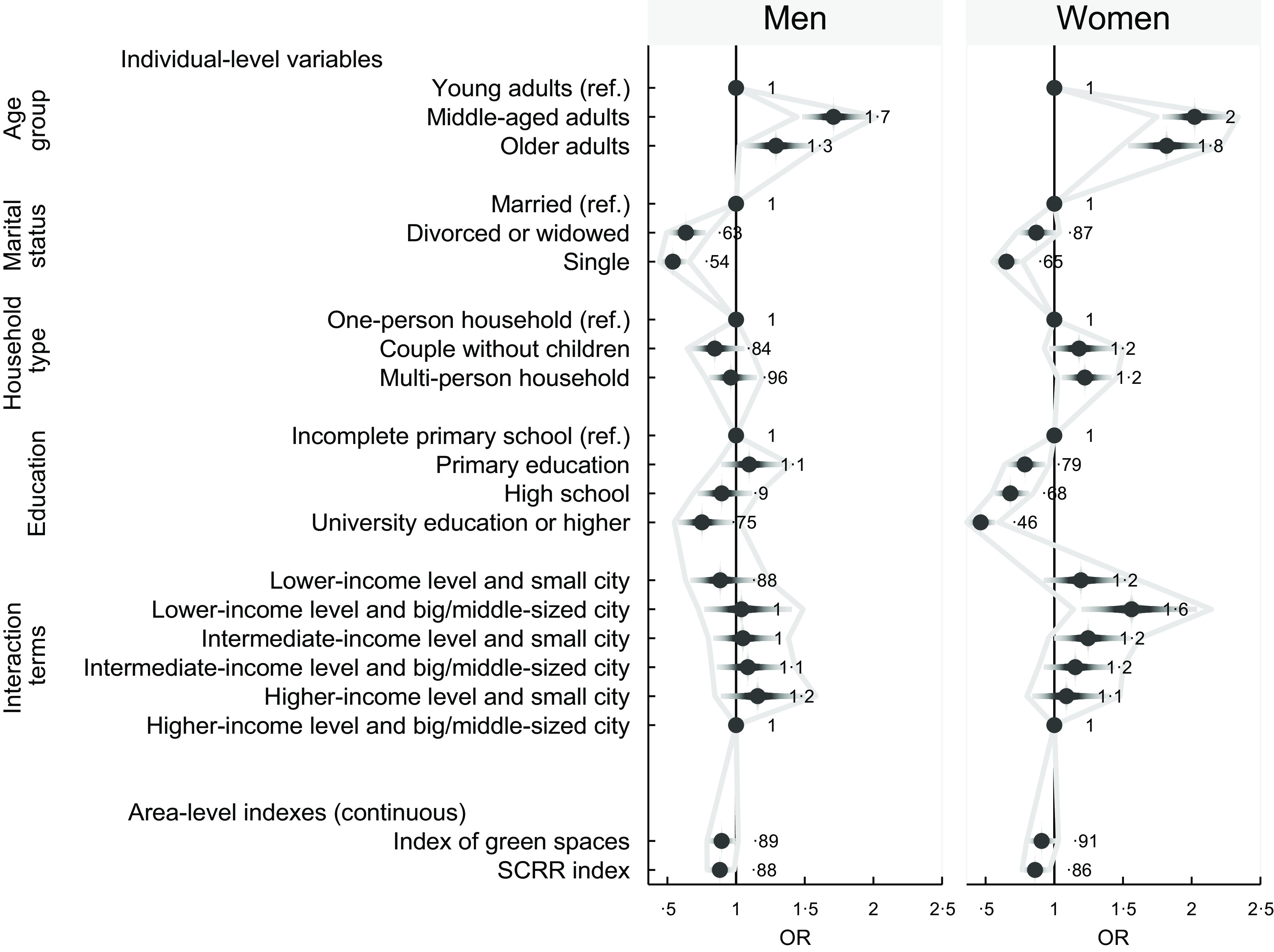
Association between socio-environmental factors and obesity in Argentina: OR and CI estimated by sex-specific models. Results from two-level logistic regression models with obesity (yes/no) as outcome, and provinces as clustering variable (random effect), adjusted by level of physical activity, tobacco, and fruit and vegetable consumption. National Survey of Chronic Diseases Risk Factors, Argentina, 2018. SCRR, SCRR, socially constructed recreational resources

Participants (in the whole sample, regardless of sex) were homogeneously distributed across the categories of normal weight (30·6 %), pre-obesity (34·6 %) and obesity (33·3 %), with just 1·5 % of people with underweight (Table 1). Underweight was more prevalent in younger groups, and especially among young women (2·9 %). Over 65 % of the total participants (71·4 % in men and 65·2 % in women) had a BMI of 25 or more (pre-obesity or obesity) (Table 1). Figure 1 presents the weight status distribution by sex and age group. As this figure shows, the most frequent category of the weight status was pre-obesity (BMI 25–29·9) in men (all age groups) and obesity (BMI ≥ 30) in women (middle-aged and older age groups). In the whole sample, obesity prevalence was 33·3 %. Both in men and women, the higher values were concentrated in middle-aged groups (Table 1). However, there were differences in obesity prevalence between sexes, mainly among older adults (41·9 % *v* 36·9 % in women and men aged 65 years or more, respectively) (Table 1). Figure 2 depicts the adjusted OR of obesity estimated by the female- or male-only multilevel models (for all ages together). Middle-aged adults were 1·7 to 2 times more at risk of having obesity than young men or women. The elderly category also showed a similar OR (1·8) in women. Other individual-level characteristics such as living as a couple or in a multi-person household, and living in a small city with a lower-income level, showed opposite tendencies in women (direct association with obesity) and men (inverse association). A lower obesity risk of being divorced/widowed or single (*v*. married) was found. In both sexes, there was a social gradient of the education level (people who are more advantaged in terms of education had lower obesity risk than those who are less advantaged); however, the risk reduction at higher educational level was stronger in women than men. Furthermore, higher SCRR and green spaces indexes were inversely associated with obesity in both groups (Fig. 2).

Although Figure 2 shows that differences between sexes, without discrimination by age group, are slight, interesting results emerged when analyses were performed by sex and age groups. Crude and adjusted OR estimates by multilevel models stratified by age group among men and women separately are displayed in Tables 2 and 3. After controlling for lifestyle covariates, it was found that compared to married men, single (in all age groups) and divorced/widowed men (in the older and middle-aged groups) had lower risk of obesity (Table 2). In the female-only model (Table 3), the association between obesity occurrence and being single (lower risk compared to married) was significant among young and middle-aged women. There was a direct association between obesity and living in a multi-person household (*v*. one-person household) in middle-aged women (OR = 1·26, p = 0·042). Besides, a social gradient by education level was accentuated in the young women group (Table 3). In particular, higher education showed a significant inverse association with obesity in all the age groups for women, as well as in middle-aged men (Tables 2 and 3). The association of income level, coupled to geographical location (interaction term), with obesity was significant only for women (Table 3). Specifically, we observed that the income level factor is not independent of geographical location among young and middle-aged women; in these population groups, a lower-income level coupled with a highly urbanised context seems to be an unfavourable scenario related to the obesity outcome. An inverse association with obesity was observed for SCRR and green space indexes (lower risk as score increases), which was significant in young groups of both sexes (Tables 2 and 3).

**Table 2:** Multilevel models examining the association of individual-level and contextual characteristics with obesity among men by age group. National Survey of Chronic Diseases Risk Factors, Argentina 2018

				Male adult population of Argentina age-stratified multilevel models†								
	Young adults Model I				Middle-aged adults Model II				Older adults Model III			
Covariable (fixed effects)	OR_crude_ ‡	OR_adj_ §	95 % CI	*P*-value	OR_crude_ ‡	OR_adj._ §	95 % CI	*P*-value	OR_crude_ ‡	OR_adj._ §	95 % CI	*P*-value
Individual-level social variables												
Marital status												
Married	1	1	–	–	1	1	–	–	1	1	–	–
Divorced or widowed	0·91	0·95	0·66, 1·36	0·768	0·63**	0·64**	0·47, 0·85	0·003	0·55**	0·52**	0·33, 0·81	0·004
Single	0·47***	0·52***	0·43, 0·62	< 0·001	0·65**	0·66*	0·47, 0·91	0·011	0·54*	0·55 *	0·31, 0·98	0·041
Household type:												
One-person household	1	1	–	–	1	1	–	–	1	1	–	–
Couple without children	0·88	0·94	0·66, 1·33	0·734	0·78	0·74	0·51, 1·05	0·093	0·87	0·81	0·50, 1·31	0·394
Multi-person household	1·09	1·15	0·90, 1·47	0·262	0·84	0·86	0·64, 1·15	0·310	0·79	0·78	0·51, 1·19	0·250
Education:												
Incomplete primary education or lower	1	1	–	–	1	1	–	–	1	1	–	–
Primary education	1·06	0·12	0·75, 1·66	0·579	0·96	1·00	0·74, 1·36	0·981	1·07	1·11	0·82, 1·51	0·499
High school	0·89	0·97	0·65, 1·46	0·901	0·77	0·75	0·55, 1·08	0·129	0·87	0·87	0·59, 1·28	0·481
Higher education	0·72	0·78	0·49, 1·23	0·284	0·66*	0·68*	0·47, 0·99	0·049	0·86	0·83	0·50, 1·40	0·490
IL × geographic location:												
Higher IL, big or middle-sized city	1	1	–	–	1	1	–	–	1	1	–	–
Lower IL, small city	1·08	1·06	0·72, 1·57	0·745	0·75	0·70	0·45, 1·07	0·097	0·97	0·93	0·48, 1·81	0·833
Lower IL, big or middle-sized city	1·04	1·04	0·69, 1·57	0·841	1·39	1·35	0·85, 2·13	0·203	0·50	0·55	0·25, 1·19	0·131
Intermediate IL, small city	1·34	1·30	0·94, 1·82	0·113	1·00	0·93	0·66, 1·31	0·694	0·77	0·74	0·47, 1·18	0·205
Intermediate IL, big or middle-sized city	1·30	1·28	0·92, 1·79	0·145	1·03	1·01	0·72, 1·44	0·932	0·79	0·80	0·50, 1·26	0·335
Higher IL, small city	1·10	1·09	0·74, 1·60	0·653	1·18	1·14	0·78, 1·67	0·500	1·28	1·26	0·74, 2·14	0·393
Area-level variables												
SCRR index (continuous variable)	0·84**	0·84**	0·74, 0·95	0·008	0·88	0·86	0·74, 1·01	0·075	0·97	0·97	0·81, 1·15	0·712
Index of green spaces (continuous variable)	0·89	0·86*	0·75, 0·98	0·027	0·91	0·89	0·74, 1·06	0·192	1·04	1·00	0·81, 1·25	0·964

IL, income level; SCRR, socially constructed recreational resources.

*P < 0·05, **P < 0·01, or ***P < 0·001 as levels of significance.

† Two-level logistic regression model with provinces as clustering variable (random effect).

‡ OR crude.

§ OR adjusted by level of physical activity, tobacco consumption, and fruit and vegetable consumption.

**Table 3:** Multilevel models examining the association of individual-level and contextual characteristics with obesity among women by age group. National Survey of Chronic Diseases Risk Factors, Argentina 2018

				Female adult population of Argentina age-stratified multilevel models†								
	Young adults Model I				Middle-aged adults Model II				Older adults Model III			
Covariable (fixed effects)	OR_crude_ ‡	OR_adj._ §	95 % CI	*P* value	OR_crude_ ‡	OR_adj._ §	95 % CI	*P* value	OR_crude_ ‡	OR_adj_ §	95 % CI	*P*-value
Individual-level social variables												
Marital status:												
Married	1	1	–	–	1	1	–	–	1	1	–	–
Divorced or widowed	1·05	1·08	0·85, 1·37	0·534	0·82	0·82	0·67, 1·01	0·069	0·91	0·86	0·60, 1·23	0·409
Single	0·60***	0·62***	0·53, 0·73	< 0·001	0·79	0·78*	0·61, 0·99	0·044	0·72	0·67	0·43, 1·06	0·087
Household type:												
One-person household	1	1	–	–	1	1	–	–	1	1	–	–
Couple without children	1·16	1·14	0·81, 1·62	0·450	1·26	1·26	0·93, 1·71	0·133	1·19	1·14	0·74, 1·74	0·543
Multi-person household	1·23	1·23	0·95, 1·60	0·120	1·20	1·26*	1·01, 1·57	0·042	1·14	1·17	0·92, 1·49	0·205
Education:												
Incomplete primary education or lower	1	1	–	–	1	1	–	–	1	1	–	–
Primary education	0·49***	0·47***	0·33, 0·65	< 0·001	0·83	0·86	0·66, 1·13	0·292	0·82	0·81	0·64, 1·03	0·092
High school	0·37***	0·36***	0·26, 0·51	< 0·001	0·73*	0·76	0·57, 1·02	0·071	0·91	0·92	0·68, 1·23	0·562
Higher education	0·27***	0·25***	0·17, 0·36	< 0·001	0·44***	0·47***	0·35, 0·65	< 0·001	0·67*	0·64*	0·44, 0·92	0·015
IL × geographic location:												
Higher IL, big or middle-sized city	1	1	–	–	1	1	–	–	1	1	–	–
Lower IL, small city	1·35	1·25	0·86, 1·83	0·237	1·13	1·13	0·78, 1·65	0·511	1·03	1·15	0·66, 2·00	0·609
Lower IL, big or middle-sized city	1·63*	1·56*	1·06, 2·30	0·024	1·61*	1·67*	1·12, 2·49	0·012	1·48	1·64	0·91, 2·95	0·099
Intermediate IL, small city	1·42*	1·35	0·96, 1·91	0·087	1·31	1·35	0·99, 1·85	0·058	1·10	1·13	0·75, 1·70	0·551
Intermediate IL, big or middle-sized city	1·36	1·27	0·90, 1·81	0·175	1·11	1·12	0·81, 1·54	0·489	1·03	1·08	0·72, 1·61	0·713
Higher IL, small city	1·48*	1·41	0·95, 2·08	0·086	1·02	1·02	0·71, 1·46	0·930	0·93	0·94	0·58, 1·52	0·798
Area-level variables												
SCRR index (continuous variable)	0·77***	0·76***	0·68, 0·85	< 0·001	0·89	0·90	0·77, 1·04	0·163	0·97	0·97	0·85, 1·11	0·674
Index of green spaces (continuous variable)	0·86**	0·85**	0·76, 0·96	0·007	1·03	1·02	0·87, 1·21	0·777	0·87	0·86	0·73, 1·02	0·094

IL, income level; SCRR, socially constructed recreational resources.

*P < 0·05, **P < 0·01, or ***P < 0·001 as levels of significance.

† Two-level logistic regression model with provinces as clustering variable (random effect).

‡ OR crude.

§ OR adjusted by level of physical activity, tobacco consumption, and fruit and vegetable consumption.

## Discussion

This study provides an updated and comprehensive picture of the obesity burden and its socio-environmental determinants in the adult population of Argentina, considering the multilevel structure of the national information. In our work, the 2018 obesity prevalence was about 33 %, with figures higher than 40 % for middle-aged groups (both sexes) and older women. For 2018, our findings indicate that the association of social and environmental factors with the obesity occurrence differ considerably across age and sex groups. In males, marital status emerges as an individual-level factor associated with obesity, and a high education level showed a significant association for the middle-aged group. In women, our findings suggest that the education level, having a multi-person household, and living in a highly urbanised context with a lower-income level are key factors associated with obesity occurrence, with differences by age groups. Contextual indexes of SCRR and green spaces were associated with obesity, specifically in the younger groups of both sexes.

The 2018 CDRF survey collected both self-reported and measured anthropometric data of the adult population of Argentina. Compared to previous statistics from the CDRF survey about self-reported BMI, increasing rates of obesity were observed since 2005^(30)^. The 2018 CDRF report indicates that the prevalence of obesity obtained from measured data was about 7 % point higher than those based on self-reported BMI^(30)^. The high levels of obesity estimated for Argentina in 2018 are consistent with the growing burden of obesity reported in the Latin American region^(3,14)^. The changes in the nutritional profile of the Latin American populations in the last decades have largely been attributed to the process of the nutrition transition^(3)^, which occurred in parallel with several socio-economic and demographic shifts (e.g. changes linked to the urbanisation and globalisation processes) in most regions of the world^(8)^. Interestingly, a recent study in Argentina reports that sociodemographic factors (such as urbanisation and poverty level) play a major role in shaping diverse nutritional profiles across the territory, which configure a complex and heterogeneous socio-nutritional scenario^(17)^.

Overall, existing studies about the association between marital status and overweight indicate that the former appears to influence obesity more strongly among men than women^(31,32)^. The lower obesity risk in single men has been reported by other studies^(19,33,34)^. This could be related to the lifestyle of married men that may lead to a more stable eating pattern, compared to unmarried people^(31)^. Besides, a larger body size is likely to be valued as a sign of physical dominance and prowess for men^(35)^, if we consider the expected social roles for men entering marriage in some social groups. It is important to note that the lower obesity risk observed in single men in our study was also present among single females, particularly in the younger groups. In this case, this finding may reflect certain body weight norms and expectations in our society linked to the female beauty ideal, more solid at certain stages of life. An exaggerated thin body ideal has been recognised as a distinctive characteristic of the Argentine population^(36)^, especially among women.

We also found a significantly lower risk for the divorced/widowed category in the middle-aged and elderly men groups (*v*. married). This could be explained, in part, by the fact that marital disruption (i.e. being widowed or divorced) has been associated with poor physical health outcomes^(32)^, including weight loss linked to psychosocial frailty or high-risk alcohol or tobacco consumption. Additionally, it has been proposed that the marital role provides support and resources which may influence eating and physical activity habits^(31)^. Thus, the lower obesity risk observed for divorced/widowed men older than 44 years of age (*v*. married ones) may indirectly reflect their potential lack of social support as observed, for example, in eating habits. The importance of social support for health is well recognised^(37)^, as well as the role of women as the ones mostly responsible for food preparation in families^(35)^. In Argentina, a national study on older adults highlights that women have a high physical and psychological burden associated with such activities as the care of elderly or family members^(38)^.

Education is a recognised individual-level socio-economic factor related to obesity risk. Overall, there is a general agreement that the relationship between education and obesity is often more consistent among women^(34,39,40)^. Additionally, we found a stronger educational gradient in the obesity burden among the younger group of Argentine women. This finding is in line with previous studies carried out in this country in 2005, which reported a lower obesity prevalence with better education level among women aged 20–49 years^(41)^. Also in other Latin American countries, lower obesity prevalence was observed in women with higher education among this age group^(42,43)^. Particularly from a longitudinal study, a reversal of the inverse association between education level and obesity risk during ageing was observed, especially in women. These authors propose that women with higher education put more effort than men into controlling their body weight, to fit themselves in the labour market and to reach a high social position; then, approaching old age, women try to get rid of the social pressures towards thinness^(44)^. A sociocultural pressure on females to achieve the desired body image, especially among women of high socio-economic status suggested by other authors^(45)^, can explain, in part, our findings in Argentina.

Since education has a role as part of the complex phenomenon of socio-economic stratification, another aspect to considerer is that people with higher education may have greater job opportunities and, therefore, better access to physical activity facilities and healthy eating. Furthermore, education can be interpreted as a proxy for ‘health literacy’^(39,40)^, which could improve an individual’s capacity to adequately address health-related issues, including overweight^(40,46)^. These could be underlying mechanisms that explain, in part, the relationship observed between obesity and higher education, especially in middle-aged men and elderly women.

It has been highlighted that socio-economic inequalities within cities in developing countries are high and affect the social distribution of health outcomes^(47)^. In Argentina, previous evidence indicates that the socio-economic patterning of chronic disease risk factors, including obesity, was modified by urbanicity^(48)^, and that poverty and urban scale are associated contextual variables influencing the distribution of non-communicable diseases mortality^(22)^. In our study, a higher risk of obesity was observed among women under 65 years of age living in big or middle-sized cities with a low family income level. Income generally reflects the availability of economic and material resources and, thus, it influences dietary quality^(49)^. These results may reflect poor access to a healthy diet and low opportunities to adopt healthy behaviours, in women with a low-income level, especially if they live in urbanised areas. Interestingly, Oliveira *et al.* ^(50)^ indicated that there are several aspects (physical, economic, political and sociocultural) of the environmental factors that would independently affect men and women, and that obesogenic influence of the environment may differ in small and medium-sized cities, when compared to large cities. According to the WHO, urban poverty and unhealthy living conditions themselves are social determinants of health that can affect disproportionately certain vulnerable subgroups such as women^(51)^. Moreover, considering their potential family caregiver role^(38)^, especially under unfavourable economic conditions, Argentine women may reduce their time for the care of their own health (impacting on their weight status). This could also explain, in part, the higher obesity risk in middle-aged women living in a multi-person household that we observed in our results.

Considering environmental factors, there is suggestive evidence about the relationship between green spaces and weight status or obesity-related health indicators. Several studies found evidence that these relationships varied by factors such as age and socio-economic status, although the mechanisms through which green spaces may influence health are not completely understood^(52)^. Evidence suggests that there is a link between green spaces and obesity, as the former would offer enhanced opportunities for physical activity^(52,53)^, even within urbanised contexts^(54)^. However, the findings on this matter are not conclusive, especially in developing countries. In Argentina, agriculture and tourism are motors of regional economic development; agricultural and touristic activities usually take place in locations where natural resources are highly available. Thus, we additionally argue that the environmental indicator used here may represent proxy variables of socio-economic conditions at the macro-level.

From a gendered perspective, MacBride-Stewart *et al.* ^(55)^ identify key dimensions to consider in the study of the interconnections between health and nature, including accessibility, availability and usability of green spaces, as well as the boundaries (symbolic/material) that construct differential relationships between natural spaces, sex and health^(55)^. Consistently, another work^(56)^ also indicates that the perception and use of green spaces, as well as green space attributes, can explain the different associations with BMI that the authors observed among age- and sex-specific adult groups. Given our finding of an association between area-level variables and obesity occurrence, those variables may also be relevant aspects to explain age group differences in Argentina.

Particularly, the role of the contextual conditions summarised in the SCRR index (such as urban aesthetics/urban heritage, cultural amenities, shopping malls and sports centres) is underexplored in obesity research. However, the notion of SCRR could be closely related to the idea of built environments, defined as the human-modified space in which people conduct their daily lives; their influence on obesity has been more extensively studied^(57)^. In general, there is consensus that the built environment plays a key role as a barrier or enabler to physical activity and as a mediator in access to healthy food^(54,58)^. Interestingly, an exhaustive review from the perspective of developing countries^(50)^ concludes that health-promoting built environments can have a profound influence not only on the population levels of physical activity but also on its well-being and equity – both socio-economic and age-sensitive. Thus, our findings of a lower obesity risk associated with contexts with greater SCCR in young people could be related to a mechanism mediated by the availability, perception and use of sports centres or other amenities that were part of the SCRR index. In a Brazilian study on obesity, the authors reinforce the idea that a favourable decrease in ‘obesogenic’ traits in the urban environment is possible when physical structures are planned to facilitate physical activity^(50)^. Alternatively, we propose that the distribution of the SCRR variable could depict a sort of ‘regionalisation’ (geographical division) underlying socio-economic and cultural characteristics of the populations or could represent different patterns of land use. Consistent evidence showed that a better mix of land use (residential, commercial, institutional, industrial, recreational and agricultural) is generally associated with less obesity burden^(12)^, although there is a general lack of insight into the pathway by which land use mix impacts obesity. Further research would be useful in these regards to better understand the observed age differences.

A major strength of this research is its updated source of data and large sample size based on a rigorous probabilistic sampling design which ensures its national representativeness. Indeed, the matching between the age group distribution in our sample and in the national population projections for 2018 by the INDEC is notable. Besides, as far as we know, this is the first study that uses measured anthropometric data to identify social inequalities in obesity distribution from the most recent CDFR survey. Moreover, it provides an analytical example of how the multilevel epidemiological framework can be used in this field of study. However, there are limitations to consider. First, we know that our study was based on an urban setting and, thus, rural populations were not represented. However, about 91 % of the Argentine population is living in urban areas^(22)^. Second, the reliability of income measures may be debatable in developing countries^(59)^, although several questions regarding household income were designed to minimise response bias in the CDRF. Also the use of BMI as a measure of obesity could be discussed, since this index should be preferably accompanied by other body composition measures in some specific population groups, such as athletes and the elderly. However, the WHO recognises that BMI provides the most useful population-level measure of overweight and obesity^(1)^. Finally, considering that exposure measurement error may occur in the characterisation of green spaces in epidemiological studies^(53)^, and that other unmeasured macro-level factors may affect conclusions, the interpretation of macro-contextual association measures should be conservative.

To conclude, our findings show high levels of obesity in Argentina in 2018 and an unequal distribution of their socio-environmental determinants. Specific social and environmental factors seem to operate differently according to sex and age groups in this country. Thus, comprehensive interventions against the obesity epidemic addressing gender inequalities and socio-environmental disadvantages at each stage of life are needed. Our results also showed a favourable relationship (inverse association) between obesity risk and contextual characteristics (such as availability of green spaces) among the younger groups. This target population could be considered in the design of interventions aimed at achieving healthy environments. Finally, further interdisciplinary research on sociocultural pathways linked to obesity within urban contexts in developing countries is needed.
